# From sequence space to ecological function: microbiome-derived antimicrobial peptides as community effectors and therapeutic leads

**DOI:** 10.1042/EBC20250036

**Published:** 2026-09-02

**Authors:** Linda Boniface Oyama

**Affiliations:** Institute for Global Food Security, School of Biological Sciences, Queen’s University of Belfast, 19 Chlorine Gardens, Belfast, BT9 5DL, UK

**Keywords:** antimicrobial peptides, ecology, metagenomics, microbial competition, microbiome, peptide antibiotics

## Abstract

Antimicrobial peptide research has long centred on host defence molecules, yet microbiomes themselves encode a diverse and increasingly important repertoire of peptide-based antimicrobials. These microbiome-derived antimicrobial peptides include bacteriocins, ribosomally synthesised and post-translationally modified peptides, cryptic short open reading frame-encoded peptides, embedded antimicrobial regions within larger proteins, and selected peptide antibiotics recovered from human, animal, plant and environmental microbiomes. Recent advances in genome mining, metagenomics, and machine learning have greatly expanded the scale of discovery, moving the field from a handful of landmark exemplars to large candidate catalogues spanning the global microbiome. In the clearest cases, these molecules are not only anti-infective leads but ecological effectors: they mediate microbial competition, enforce colonisation resistance, and influence community structure within densely occupied niches. The present review synthesises the field across discovery classes, microbiome sources, ecological roles, and translational bottlenecks, emphasizing a central limitation of the field: candidate catalogues are expanding at extraordinary scale, while evidence for native expression, producer assignment, ecological function, and *in vivo* relevance remains limited for the vast majority of predicted molecules. Progress will depend on workflows that connect sequence level prediction to biological context through expression support, producer assignment, community level validation, and perturbation-based approaches that distinguish ecological association from causal function. Microbiome-derived antimicrobial peptides are best understood not only as promising therapeutic leads, but also as molecular mediators of microbial social life whose ecological origins are central to their interpretation and future application.

## Introduction

Research on antimicrobial peptides (AMPs) has long focused on host defence peptides, particularly defensins, cathelicidins, and related innate immune effectors [1–3]. Since the mid 2010s, increasing attention has turned to peptides produced not by the host, but by members of the microbiome itself [4–9]. This shift changes both the source of discovery and the biological framework within which these molecules are interpreted. Rather than being understood primarily as host defence factors, microbiome-derived antimicrobial peptides can be viewed as products of microbial competition within densely populated and contested ecosystems [9,10].

Microbiome-derived antimicrobial peptides are defined here as peptide antimicrobials identified from microbiome datasets, encoded by microorganisms recovered from defined microbiomes, or characterised in ways explicitly grounded in microbial community context. This excludes host-derived antimicrobial peptides, broad non-peptidic microbiome metabolites, and probiotic effects that cannot be attributed to a defined peptide. The distinction matters because microbiomes are not simply rich environments from which useful molecules may be isolated [4,11]. The community context is itself part of what gives these peptides biological and translational significance [11,12].

Microbial communities are shaped not only by cooperation and metabolic exchange, but also by chemical antagonism, territorial exclusion, and competitive niche defence [12,13]. Within such settings, antimicrobial peptides can function as ecological effectors [9,12,14]. They may contribute to colonisation resistance, suppress competitors, and influence which organisms persist within a niche. Well-known examples, including lactocillin from the vaginal microbiota [4], lugdunin from the nasal microbiota [9], archaeasins from archaeal proteomes [15], and antimicrobial candidates recovered from the rumen microbiome [6,16,17], show that microbiome-associated peptide discovery is neither confined to a single microbiome type nor to a single discovery strategy.

**Figure 1:**
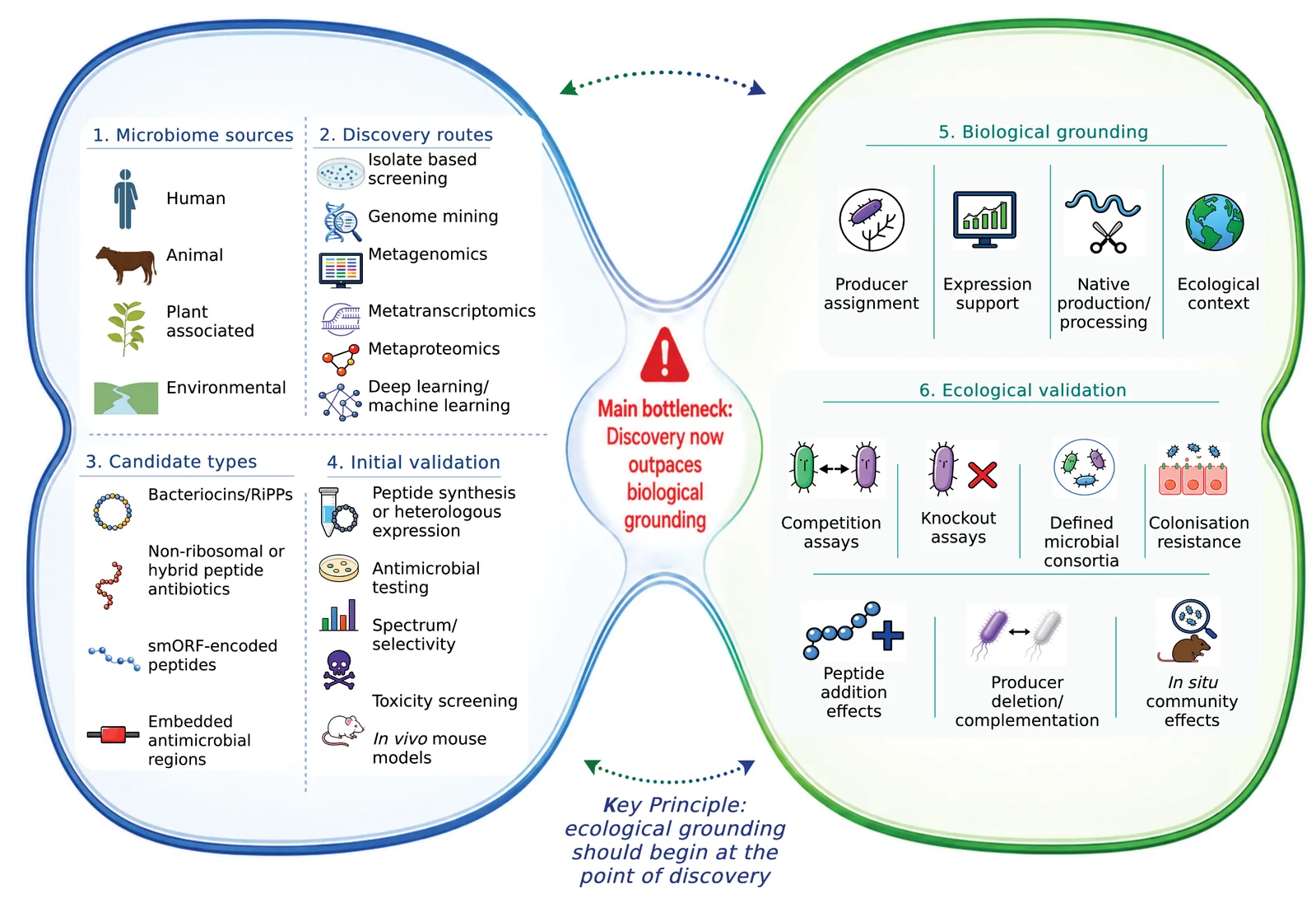
From microbiome sequence space to ecological function: a framework for the discovery, validation, and ecological grounding of microbiome-derived antimicrobial peptides The left zone (blue) represents the translational discovery and validation pipeline, encompassing microbiome sources (panel 1), discovery routes (panel 2), candidate types (panel 3), and initial validation (panel 4). The right zone (green) represents the biological grounding and ecological validation steps currently absent from most discovery pipelines, including confirmation of producer identity, expression support, native processing, and ecological relevance (panel 5), and community level validation through competition assays, defined consortia, colonisation resistance testing, perturbation approaches, and *in situ* community relevance (panel 6). The central bottleneck marks the point at which most current pipelines terminate. Dashed arrows and the annotation indicate that ecological grounding should inform all stages of discovery, not only the final validation steps. The goal of an integrated pipeline is to connect sequence-level prediction to demonstrated causal ecological function. Created in BioRender; Oyama, L. (2026) https://BioRender.com/nm6tq53

Recent advances in metagenomics, computational mining, and machine learning have transformed the scale of discovery [5,7]. Evidence across the field remains uneven, ranging from computationally predicted candidates and *in vitro* validated peptides [8] to a much smaller number of molecules supported by ecological association, *in situ* expression, or direct *in vivo* evidence [9]. Large datasets can now yield hundreds or even hundreds of thousands of candidate peptides [5,7], yet prediction has moved faster than validation. Microbiome-derived antimicrobial peptides are therefore increasingly visible as therapeutic leads, which indeed they are, while their ecological functions, natural expression patterns, and community level roles often remain incompletely resolved [18]. They are most usefully understood through both their ecological roles in microbial communities, including as mediators of microbial competition [12], and their potential as therapeutic leads, particularly as computational discovery continues to outpace functional validation [19]. This position shapes how these molecules are interpreted, why they are thought to exist, and how they may ultimately be applied (Figure 1). Understanding the ecological context of microbiome-derived antimicrobial peptides matters not only for explaining why these molecules exist and how they function in microbial communities, but also for improving how candidate peptides are prioritised for therapeutic development. Without biological grounding, sequence-level discovery risks identifying molecules that are interesting in principle but poorly resolved in terms of native expression, ecological function, and translational relevance.

## From foundational microbiome mining to large scale predictive discovery

### Foundational microbiome mining, 2010 to 2018

The modern field of microbiome-derived antimicrobial peptide discovery did not begin with machine learning, nor did it emerge only once uncultured communities became accessible at scale. Its early development was already methodologically mixed, combining isolate-based work [9], genome mining, metagenomic screening [4,6,17], and ecological interpretation [6,9]. A defining early landmark was the systematic analysis of biosynthetic gene clusters in the human microbiome by Donia and colleagues [4], which showed that human associated microbial communities contain widespread biosynthetic capacity for peptide natural products, including thiopeptides, and led to the isolation of lactocillin from a prominent member of the vaginal microbiota. That work [4] established the human microbiome as a credible reservoir of previously underappreciated chemical diversity rather than merely a backdrop for host interactions.

A second formative example came from the nasal microbiome. Zipperer and colleagues identified lugdunin, a cyclic peptide antibiotic produced by *Staphylococcus lugdunensis*, and showed that its presence correlated with impaired *Staphylococcus aureus* colonisation [9]. Lugdunin is particularly instructive because it linked antimicrobial discovery directly to ecology. A resident microbiota member was shown to deploy peptide-based chemistry in a way that could influence who occupies a host associated niche. Ecological grounding in this early phase of microbiome-derived antimicrobial peptide discovery was not confined to the nasal microbiome. In the gut, microcins produced by *Escherichia coli* Nissle 1917 were shown to mediate competition among enterobacteriaceae in the inflamed gut, providing direct evidence that microbiome-derived peptide antimicrobials can shape bacterial competition under ecologically relevant conditions *in vivo* [10]. On the skin, coagulase-negative staphylococci resident on healthy human surfaces were shown to produce AMPs that selectively suppress *S. aureus*, and AMP-producing strains were deficient in patients with atopic dermatitis, suggesting a causal role for community-encoded peptides in colonisation resistance at epithelial surfaces [14]. These studies showed that peptide-based chemical competition among microbiome members was not a curiosity of a single niche, but a recurring feature of host-associated communities.

This early phase of microbiome-derived antimicrobial peptide discovery was not restricted to human body sites. Work on non-human microbiome-derived peptides, including early studies from the rumen, also formed part of the field’s expansion beyond more conventional isolate centred bacteriocin discovery. The 2017 rumen microbiome study by Oyama and colleagues showed that a non-human microbiome could also serve as a productive discovery space for novel antimicrobial peptides [6]. Using functional screening and computational approaches, that study identified 181 potentially novel AMPs from a rumen bacterial metagenome and validated lead candidates, including Lynronne peptides, with activity against clinically important multidrug resistant pathogens [6]. Together, these early studies established three points that still shape the field. First, microbiomes harbour peptide-based antimicrobial potential that is not well captured by classical antibiotic discovery frameworks [4,6,11]. Second, microbiome-derived molecules can function as ecological effectors within native communities, not merely as *in vitro* curiosities, with demonstrated roles in colonisation resistance and competitive exclusion [9,10,20]. Third, promising discovery sites extend well beyond the most heavily sampled human niches [4,6,9,21].

### Platform expansion, 2019 to 2022

From 2019 to 2022, the field broadened its discovery pipelines rather than undergoing a single methodological break. Metagenomic resources expanded, computational screening became more systematic, and candidate generation moved beyond the most familiar bacteriocin classes. Studies increasingly combined sequence-based prediction with peptide synthesis, phenotypic testing, and limited functional characterisation. The main transition was not simply from cultured to uncultured systems, but from targeted discovery of individual molecules towards more systematic and scalable candidate generation [5]. Ecologically grounded work had already demonstrated that microbiome-encoded peptides could function as community effectors in native host-associated settings, and this understanding deepened during the same period. The microbiome-shaping roles of bacteriocins across human body surfaces, including their capacity to mediate competitive exclusion and influence community composition, were synthesised in a 2021 review that also mapped the expanding landscape of biosynthetic gene clusters accessible through metagenomic screening [18]. A 2020 study identified *Staphylococcus capitis* on the skin as a commensal that selectively suppresses *Cutibacterium acnes* through the synergistic action of secreted phenol-soluble modulin AMPs, reinforcing the idea that community-encoded peptides contribute to colonisation resistance at epithelial surfaces [22].

### Large scale predictive mining, 2023 upwards

The most recent phase has been marked by a dramatic increase in discovery scale. Deep learning and related computational approaches can now mine human and global microbiome datasets for candidate antimicrobial peptides at volumes that would have been impractical only a few years earlier [5,7]. A key milestone was the 2022 study by Ma and colleagues, which applied deep learning to human gut metagenomic data, identified 2349 candidate AMPs, and validated lead peptides in a murine model of bacterial lung infection, demonstrating that machine learning pipelines could move from sequence space to *in vivo* activity within a single study [5]. Santos-Júnior and colleagues used machine learning across 63,410 metagenomes and 87,920 prokaryotic genomes to build AMPSphere, a catalogue of more than 863,000 non-redundant peptides from the global microbiome, of which 100 were synthesised and tested, with 79 showing antimicrobial activity and three demonstrating *in vivo* efficacy [7]. The study also found that AMP biosynthetic potential varies by habitat and that predicted peptides showed activity against gut commensal bacteria [7], raising the possibility that many AMPSphere candidates function as ecological effectors within their native communities.

Most recently, Chen and colleagues extended metagenomic AMP mining to ancient gut microbiomes preserved in human coprolites, developing the AMPLiT tool to screen pre-industrial microbial communities and identifying 160 candidates, of which 90% of those synthesised showed *in vitro* activity, with the majority attributed to *Segatella copri*, a gut commensal abundant in ancient populations but depleted in modern Westernised microbiomes [23].

This predictive power has transformed the field, but it has also exposed a major tension. Discovery scale has increased faster than direct biological grounding [5,7,8]. Many predicted peptides remain unlinked to expression *in situ*, to a defined producer under native conditions, or to a demonstrated ecological role [5,7,8], and even where producer assignment or transcriptional support has been achieved, ecological function remains unresolved in the vast majority of cases [4,6]. The principal bottleneck has therefore shifted. The challenge is no longer generating candidates but determining which among them are genuinely expressed, functionally active, and ecologically relevant under native conditions, and therefore worth sustained experimental investment [7,8,24].

## What kinds of microbiome-derived antimicrobial peptides are being found?

Any account of microbiome-derived antimicrobial peptides has to address a basic problem of classification. The field now includes molecules discovered through very different routes, from classical bacteriocin screening in cultured commensals [9,13] to large-scale prediction of short cryptic peptides in metagenomic datasets [7,8]. A rigid taxonomy would therefore be misleading. A more useful approach is to classify these molecules by biosynthetic logic while remaining explicit that the boundaries are not always neat [4,8].

The best established group remains bacteriocins and related ribosomally synthesised peptides. These are typically encoded by gene clusters that include precursor peptides, modification enzymes, transport systems, and self-protection mechanisms [18,25]. In host associated microbiomes, this category includes lantibiotic and other modified peptides produced by skin, nasal, oral, gut, and vaginal commensals [26]. Many act over narrow or intermediate spectra [12,13], which is consistent with competition among close neighbours occupying the same niche. Bacteriocins therefore remain useful entry points for understanding antimicrobial peptides as community shaping molecules rather than simply reduced forms of conventional antibiotics.

A broader and increasingly important category is the RiPP-like space, meaning ribosomally synthesised and post translationally modified peptides that extend beyond the classical bacteriocin families [27,28]. Systematic mining of human associated genomes has shown that bacteria across the skin, gastrointestinal tract, mouth, trachea, and urogenital tract encode substantial diversity of lanthipeptides and lasso peptides [26], many of which were not previously recognised as part of the antimicrobial landscape. This widens the field from a handful of canonical examples to a much broader set of chemically modified peptide scaffolds with potentially distinct spectra, targets, and ecological functions.

More recent work has pushed discovery further into the space of short open reading frames (ORFs) and cryptic peptides. The 2024 *Cell* study by Torres and colleagues treated the human microbiome as a source of previously hidden peptide antibiotics encoded in small ORFs rather than restricting discovery to recognizable biosynthetic gene cluster signatures [8]. This suggests that a substantial fraction of microbiome encoded antimicrobial potential may lie outside the classes that older genome mining frameworks were designed to detect.

A related but conceptually distinct source of microbiome-derived antimicrobial peptides comes from scanning larger encoded proteins for embedded antimicrobial regions rather than searching only for dedicated peptide-encoding genes [29–31]. Computational tools applied in sliding-window fashion across microbial proteomes or translated metagenomic sequences can identify short stretches within full-length proteins that carry AMP-like physicochemical properties, including cationic charge and amphipathic character, even when the parent protein has no recognised antimicrobial annotation. These embedded, antimicrobial regions may require proteolytic processing for release and activity, and their existence within larger proteins means they are largely invisible to biosynthetic gene cluster mining and standard smORF prediction alike. This approach has been applied to microbiome sequence data and represents a further expansion of the discoverable space beyond what any single annotation framework was designed to capture [8]. This expands the accessible peptide space considerably, but it also introduces additional uncertainty, because the native processing, release, and biological relevance of such predicted regions are in most cases entirely uncharacterised.

A small but significant category blurs any clean distinction between antimicrobial peptides and natural products. Lugdunin, the cyclic thiazolidine antibiotic produced by *S. lugdunensis* in the nasal microbiome [9], and epifadin [32], an NRPS–PKS hybrid produced by *Staphylococcus epidermidis* in the same niche, exemplify this boundary. Neither is a conventional bacteriocin, yet both function as ecological effectors: lugdunin suppresses *S. aureus* nasal colonisation, while epifadin eliminates *S. aureus* from the shared nasal habitat *in vivo*, with its short half-life potentially limiting collateral damage to mutualist community members [9,32]. Such molecules are a reminder that the chemical logic of microbial competition in native communities does not map neatly onto the taxonomic categories of natural product chemistry, and that discovery frameworks built around a single biosynthetic class may miss some of the most ecologically informative examples.

## Which microbiomes have been explored, and which remain underexplored?

The discovery landscape for microbiome-derived antimicrobial peptides is highly uneven. Human associated microbiomes dominate the literature, especially the gut [5,8,26], reflecting sequencing depth, available reference genomes, and the broader infrastructure built around human microbiome research rather than any settled conclusion that these niches are uniquely productive. The gut has therefore become the largest apparent reservoir of microbiome-derived peptide candidates, particularly in metagenomic and computational studies [5,7,8], but abundance of candidates should not be conflated with abundance of functionally expressed or ecologically important molecules. Other human body sites, including the nasal [9], skin [14], vaginal [4], and oral microbiomes [33,34], have also yielded important examples, but their significance lies more in the strength of selected ecologically grounded discoveries than in the scale of candidate generation.

Beyond the human microbiome, the field remains comparatively underdeveloped. Animal associated microbiomes, including those of livestock, such as ruminants [6,35–37], poultry [38], swine [39], and aquaculture species [40], as well as insects [41], wildlife [42], and companion animals [43], represent promising but still sparsely characterised sources of peptide antimicrobials. The same is true of plant associated microbiomes, including seed and endophytic communities [44], soil microbiomes [45], and marine and other environmental microbiomes [7], where biosynthetic potential is increasingly apparent but compound level characterisation remains limited. In many of these systems, the gap between genomic potential and functionally resolved peptide discovery appears especially wide, suggesting that current maps of microbiome-derived antimicrobial diversity are shaped at least as much by where we have looked as by where the richest chemistry actually lies.

Even within the microbiomes that have been explored, site alone may not be the most informative way to think about recurrent producer backgrounds. Recent work has suggested that lineages within Prevotellaceae may recur as candidate producer backgrounds for antimicrobial peptides [8,23], including *S. copri* from the Segatella complex, formerly *Prevotella copri* [46], and taxa assigned in earlier rumen work to *Prevotella ruminicola*, now *Xylanibacter ruminicola* [47]. Although this observation does not justify a single dominant producer lineage, it does suggest that phylogenetically recurring reservoirs may become an important theme in future work.

## Ecological relevance; what do these peptides do in microbial communities?

The strongest reason to treat microbiome-derived antimicrobial peptides as more than a drug discovery reservoir is that some already have plausible or demonstrated roles within microbial communities. In the best characterised cases, these peptides are not incidental secretions with generic antibacterial activity, but ecological effectors that help resident strains defend space, exclude competitors, and influence who can persist within a niche [9,10,14]. The clearest evidence currently points to roles in competition, colonisation resistance, and local community structuring rather than broad community wide eradication [9,10].

Several of the best known examples support this ecological framing across distinct host associated microbiomes. In the nasal microbiome, lugdunin produced by *S. lugdunensis* is associated with reduced *S. aureus* carriage [9], an association consistent with AMP-mediated competitive exclusion within the niche, as discussed above. On the skin, coagulase-negative staphylococci, particularly *S. epidermidis* and *Staphylococcus hominis*, produce antimicrobial activity against *S. aureus*, and strains with this activity are depleted in atopic dermatitis [14], suggesting that peptide mediated antagonism contributes to epithelial colonisation resistance. In the gut, microcins produced by *E. coli* Nissle 1917 provide a further example of peptide-mediated competitive exclusion *in vivo*, this time among Enterobacteriaceae in the inflamed gut [10]. Seen in this light, microbiome-derived antimicrobial peptides are not only candidate therapeutics, but molecular participants in the social lives of microbial communities.

**Figure 2:**
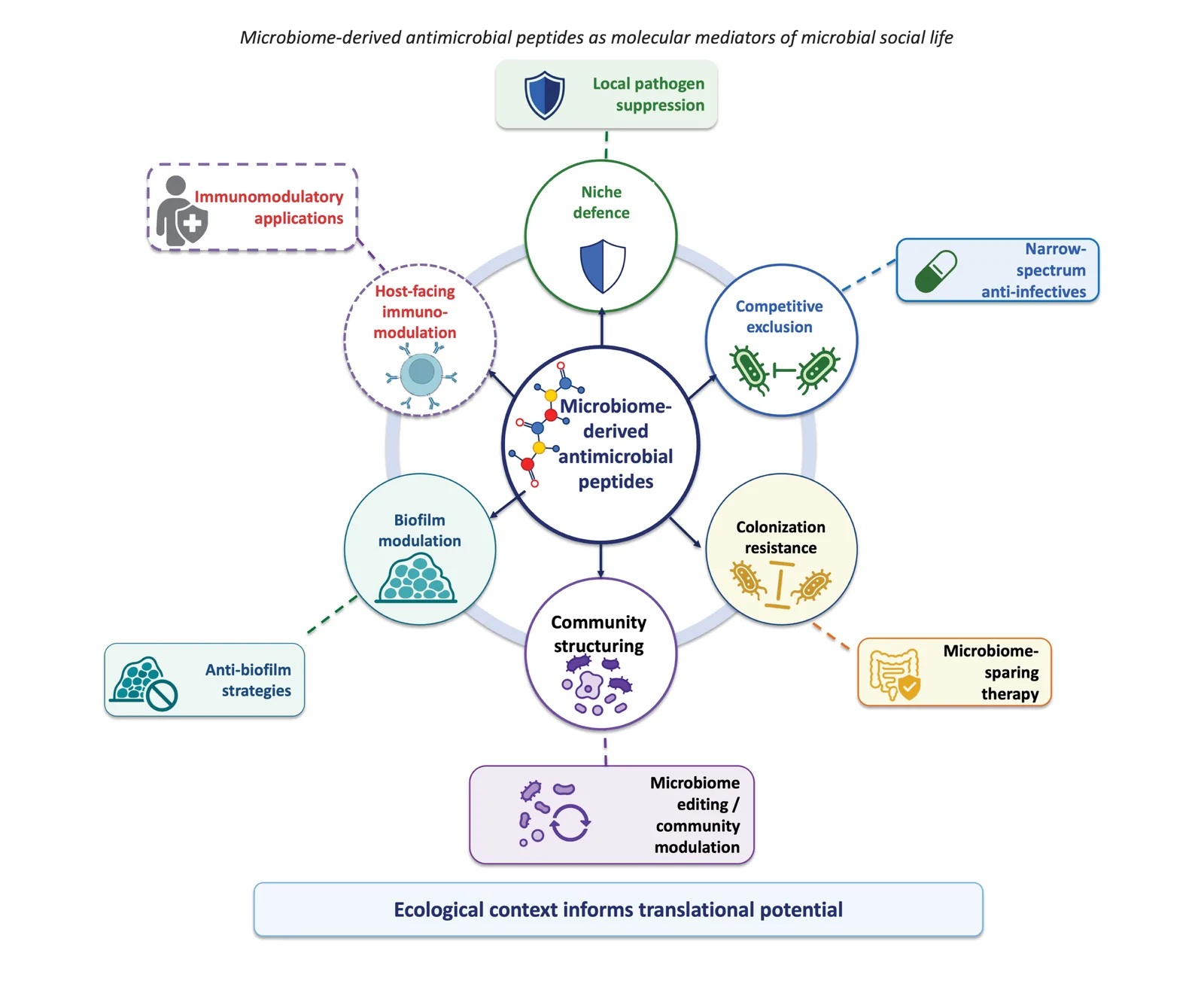
Microbiome-derived antimicrobial peptides as molecular mediators of microbial social life and sources of translational leads This figure shows the ecological and translational roles of microbiome-derived antimicrobial peptides. The inner ring shows the core ecological functions attributed to microbiome-derived antimicrobial peptides within native microbial communities: niche defence, competitive exclusion, colonisation resistance, community structuring, biofilm modulation, and host-facing immunomodulation. Each ecological function connects outwards to a corresponding translational application: niche defence to local pathogen suppression, competitive exclusion to narrow-spectrum anti-infectives, colonisation resistance to microbiome-sparing therapy, community structuring to microbiome editing and community modulation, biofilm modulation to anti-biofilm strategies, and immunomodulation to immunomodulatory applications. Dashed borders indicate categories where evidence remains emerging or less well established relative to the core ecological functions. The banner below the figure states the central interpretive principle of the present review: ecological context informs translational potential. Evidence for the ecological functions shown here is discussed in the section ‘Ecological relevance; what do these peptides do in microbial communities?’. Translational implications are assessed in the section on ‘Promise and persistent bottlenecks'. Created in BioRender; Oyama, L. (2026) https://BioRender.com/golab7r.

Taken together, these studies suggest several recurring ecological functions (Figure 2). One is niche defence, where resident strains protect occupied space against closely related or competing invaders. Another is competitive exclusion, where peptide production directly alters who can establish or persist within a community. A third is community structuring, in which peptide mediated antagonism influences relative abundance, coexistence, or succession. Some microbiome-derived peptides also show activity against biofilm formation [6,18], although the ecological significance of biofilm-modulating activity within native communities remains poorly characterised. It is important, however, not to over generalize from the best known examples. For most candidate microbiome-derived antimicrobial peptides, ecological relevance is still inferred from producer identity, niche context, or inhibition spectrum rather than demonstrated causally through knockout, replacement, or community perturbation experiments. Expression may be conditional, concentrations *in situ* may be low, and effects observed in pairwise assays may not translate cleanly into complex communities [18]. Ecology therefore remains both the field’s central strength and one of its major unresolved challenges.

A smaller but intriguing body of work suggests that some microbiome-derived peptides may influence not only competing microbes but also host facing processes, including immune responses [48,49]. For example, salivaricin 10 from *Streptococcus salivarius* has been reported to combine targeted antimicrobial activity with proimmune effects including upregulation of neutrophil-mediated phagocytosis, promotion of antiinflammatory M2 macrophage polarisation, and stimulation of neutrophil chemotaxis [48], while lactomodulin from *Lactobacillus rhamnosus* has been described as having both antimicrobial and anti-inflammatory activity [49]. However, for many microbiome-derived antimicrobial peptides, such evidence has largely come from *in vitro* systems, including cell line and immune cell assays, rather than from direct demonstration of peptide mediated immunomodulation within the native microbial community or in the original *in vivo* niche from which these peptides were identified. Immunomodulatory activity should therefore be viewed as an emerging and potentially important extension of the field, but one that remains less ecologically resolved than the better established evidence for competition, colonisation resistance, and niche defence.

## Promise and persistent bottlenecks

The translational appeal of microbiome-derived antimicrobial peptides is increasingly tangible rather than merely theoretical. They arise from densely competitive microbial ecosystems, often occupy underexplored chemical space, and in selected cases have already advanced beyond *in vitro* activity to demonstrate efficacy *in vivo*, including among candidates emerging from newer computational discovery pipelines [5,6,8]. This creates the possibility not only of new anti-infective scaffolds, but of new intervention logics, including microbiome sparing therapeutics, niche targeted suppression of colonizers, and strategies that reinforce colonisation resistance rather than simply sterilizing a site [9,14,18]. Their ecological origins may therefore be an advantage rather than a complication, especially for local or context specific applications.

A second source of optimism is the rapid expansion of discovery space itself. Deep learning, large scale metagenomic mining, and newer short ORF or hidden peptide discovery pipelines have made it possible to move well beyond the small number of classical exemplars that once dominated the field [5,7,8]. Global microbiome datasets now yield candidate sets at unprecedented scale [7], and recent work suggests that substantial fractions of microbiome encoded antimicrobial potential may have been systematically missed by earlier annotation frameworks [8]. Yet this expansion in candidate space has also sharpened the field’s central problem: discovery is becoming easier much faster than interpretation. Parallel approaches outside the microbiome mining paradigm, including generative deep learning models that design novel AMP sequences from learned chemical space [50,51] and molecular de-extinction pipelines that mine the proteomes of extinct organisms for antimicrobial sequences [52], further illustrate the breadth of computational strategies now available, though these approaches produce molecules with no microbiome origin or community context and are therefore conceptually distinct from the discovery logic the present review addresses.

The most persistent bottleneck is therefore no longer candidate generation, but biological grounding (see Figure 1). Computational pipelines can produce several thousand peptide candidates [5,7,8], yet only a small fraction are synthesised, tested, linked confidently to a producer, or shown to be expressed under biologically relevant conditions. Even fewer are supported by *in vivo* data or convincing ecological evidence within a community context. For many candidates, uncertainty remains at multiple levels at once, including whether the peptide is naturally produced, whether it is released in an active form, which organism produces it under native conditions, and whether it contributes meaningfully to competition, colonisation resistance, or community structuring. These unresolved layers create a large middle ground between sequence level possibility and ecologically resolved function.

Beyond these discovery specific issues, microbiome-derived antimicrobial peptides still face the familiar liabilities of peptide therapeutics more broadly, including proteolytic instability, short half-life, delivery challenges, possible cytotoxicity, and the difficulty of translating *in vitro* activity into durable efficacy *in vivo* [6,53]. There is also a more conceptual risk: as mining pipelines become increasingly productive, the field may begin to reward catalogue size over biological depth, leading to rediscovery, overinterpretation, or inflated claims based on weakly resolved candidates. Comparative benchmarking, dereplication, and clearer evidence hierarchies will therefore be essential if the field is to distinguish genuinely promising molecules from a rapidly growing background of plausible but still ungrounded predictions.

## Outlook and conclusion

Microbiome-derived antimicrobial peptides now sit at an important crossroads in helping to tackle the global health challenge of antimicrobial resistance (AMR). Over the past decade, the field has expanded from a few compelling exemplars to a much larger and more heterogeneous discovery space driven by metagenomics, genome mining, and machine learning. That expansion has been scientifically valuable, but it has also made it easier to lose sight of what originally made these molecules so interesting: they are products of life in densely occupied microbial communities, shaped by competition, niche defence, and the continual pressure of coexistence.

The next step is not merely to predict more peptides, but to reconnect prediction with biological context (see Figure 1). Stronger links are needed between candidate sequence, producing organism, expression under relevant conditions, ecological function, and experimentally verified activity. In practical terms, this means moving beyond catalogue generation towards integrated workflows in which metatranscriptomic and metaproteomic confirmation serve as an early filter, distinguishing candidates with genuine expression support from those that remain sequence level predictions.

Ecological validation should then move into tractable community systems. Defined microbial consortia assembled from cultivated strains representative of the native niche can be used to test whether a peptide producing strain gains competitive advantage, alters community composition, or strengthens colonisation resistance, while gnotobiotic mouse models extend the same logic into a living host context. For cultivable producers, deletion and complementation of the structural gene or biosynthetic locus should become the minimum standard for causal attribution. For uncultured producers, heterologous expression in a cultivable chassis offers a partial substitute. The key point is that ecological function must be demonstrated in community context, not inferred from pairwise inhibition alone.

This also requires better experimental readouts. Competition assays on structured or niche relevant surfaces, combined with sequence-based monitoring of community composition before and after peptide addition, producer introduction, or producer deletion, are far more informative than planktonic minimum inhibiotory concentration (MIC) data alone. Finally, peptide stability should be assessed under niche relevant conditions, including local pH, protease activity, and ionic composition, rather than only under generic physiological conditions. Together, these approaches would move the field from large candidate catalogues towards a smaller set of molecules that are expressed, causally attributed, ecologically resolved, and realistically interpretable for translation.

A second priority is to broaden the biological map of discovery. Human gut datasets currently dominate, but that dominance reflects sampling intensity as much as intrinsic productivity. Valuable peptide antimicrobials have already emerged from very different ecological settings, and there is little reason to think that currently favoured niches exhaust the relevant diversity. Non-human host associated and environmental microbiomes remain especially promising and under characterised, suggesting that the present map of microbiome-derived antimicrobial peptides still reflects where we have looked more than the full extent of what is there to be found.

Taken together, the evidence supports a simple but important conclusion. Microbiome-derived antimicrobial peptides are best understood not only as promising therapeutic leads, but also as molecular mediators of microbial social life whose interpretation depends on ecological context (Figures 1 and 2). Read only as candidates for drug development, their ecological logic becomes blurred, but read only as ecological phenomena, their translational potential is equally diminished. Understood in the context in which they evolved, as instruments of competition, coexistence, and community defence, their therapeutic promise becomes easier to interpret and more realistic to pursue.

## Summary

- Microbiome-derived antimicrobial peptides are emerging as both ecologically relevant mediators of microbial competition and promising sources of anti-infective leads for tackling AMR.
- Advances in genome mining, metagenomics, and machine learning have greatly expanded discovery, but most predicted candidates still lack strong evidence for expression, producer assignment, ecological function, and *in vivo* relevance.
- The best characterised examples show roles in competition, colonisation resistance, and community structuring, highlighting the importance of interpreting these molecules in their native ecological context.
- Future progress will require integrated workflows that connect large scale prediction for therapeutic translation with biological grounding, causal validation, and community relevant experimental systems.

## Open Access

Open access for this article was enabled by the participation of Queen's University Belfast in an all-inclusive Read & Publish agreement with Portland Press and the Biochemical Society under an agreement with JISC.

## Abbreviations

AMP: antimicrobial peptide
AMR: antimicrobial resistance
MIC: minimum inhibitory concentration
NRPS: non-ribosomal peptide synthetase
ORF: open reading frame
PKS: polyketide synthase
RiPP: ribosomally synthesised and post-translationally modified peptide
